# Comparison between Micro-Powder Injection Molding and Material Extrusion Additive Manufacturing of Metal Powders for the Fabrication of Sintered Components

**DOI:** 10.3390/ma16237268

**Published:** 2023-11-22

**Authors:** Krzysztof Siedlecki, Marcin Słoma, Andrzej Skalski

**Affiliations:** Micro- and Nanotechnology Division, Institute of Metrology and Biomedical Engineering, Faculty of Mechatronics, Warsaw University of Technology, 8 Sw. A. Boboli St., 02-525 Warsaw, Poland; krzysztof.siedlecki2.stud@pw.edu.pl (K.S.); andrzej.skalski@pw.edu.pl (A.S.)

**Keywords:** micro-powder injection molding, extrusion forming, 3D printing, powder metallurgy

## Abstract

Original compositions based on iron micro-powders and an organic binder mixture were developed for the fabrication of sintered metallic elements with micro-powder injection molding (µPIM) and material extrusion additive manufacturing of metal powders (MEX). The binder formulation was thoroughly adjusted to exhibit rheological and thermal properties suitable for µPIM and MEX. The focus was set on adapting the proper binder composition to meet the requirements for injection/extrusion and, at the same time, to have comparable thermogravimetric characteristics for the thermal debinding and sintering process. A basic analysis of the forming process indicates that the pressure has a low influence on clogging, while the temperature of the material and mold/nozzle impacts the viscosity of the composition significantly. The influence of the Fe micro-powder content in the range of 45–60 vol.% was evaluated against the injection/extrusion process parameters and properties of sintered elements. Different debinding and sintering processes (chemical and thermal) were evaluated for the optimal properties of the final samples. The obtained sintered elements were of high quality and showed minor signs of binder-related flaws, with shrinkage in the range of 10–15% for both the injection-molded and 3D printed parts. These results suggest that, with minor modifications, compositions tailored for the PIM technique can be adapted for the additive manufacturing of metal parts, achieving comparable characteristics of the parts obtained for both forming methods.

## 1. Introduction

Powder metallurgy is a technology used to fabricate construction elements by aggregating powders into a solid mass in the sintering process. This very economical process is widely used in the automotive industry, machine industry, and medical and consumer products [1]. Being more flexible than casting, extrusion, or forging techniques and minimal waste in production are the main advantages of this technology. One of the modifications of powder technology is powder injection molding (PIM), which emerged from dynamic progress in technology, merging the achievements of polymer injection molding and powder technology [2,3,4,5,6]. The idea behind PIM (or µPIM, respectively) in the macro- and microscale are the same and rely on similar steps: preparation of the composition, which consists of thermoplastic binder and functional powder, injection molding to form the final geometry, and the debinding process to remove the organic binder, followed by thermal sintering [7]. This process is categorized as near-net shape technology, allowing the fabrication of components with almost final geometry and surface finishing, reducing the time and costs of the fabrication process.

Powder technology has also stepped into the additive manufacturing area [8]. Additive manufacturing and 3D printing are terms generally used to denote a revolutionary manufacturing technology allowing the fabrication of objects layer-by-layer in an additive fashion directly from CAD files, without molding or subtractive milling [9]. The most popular technique is Fused Deposition Modeling (FDM) [10,11], which is one of the simplest approaches to additive manufacturing, where fused polymer material is deposited from a heated nozzle. On the other hand, powders are widely used in additive techniques, solidified with a liquid binder [12] or sintered with a laser and electron beam [13,14]. The most popular powder-metallurgy processes in additive manufacturing are Selective Laser Sintering (SLS), Selective Laser Melting (SLM), and Direct Metal Laser Sintering (DMLS). These layer-manufacturing processes enable generating complex 3D parts by consolidating successive layers of powder material on top of each other, by processing selected areas using thermal energy supplied by a focused laser beam. They are very popular in medical applications in the production of scaffolds with controlled porosity for the proliferation of osteoblasts in bone formation [15,16] or for dental implants [17,18].

Beside laser sintering, only a few techniques in 3D printing are directed toward the fabrication of metal or ceramic elements. Some research institutions are working to develop composites for FDM filament with metallic [19,20] or ceramic phase [21,22], with higher mechanical properties for a variety of structural and electric or bioceramic applications. Afterward, the elements are sintered, just as in the traditional powder-technology process. Another approach involves preparing the ceramic elements from slurry poured into a polymer form printed with the FDM process [23]. Extrusion free-forming (EFF), also called direct ink write (DIW), is widely used for the fabrication of ceramic elements from slurries deposited as planar layers from extrusion nozzles at room temperature (unlike the heated FDM process) [24,25,26].

This paper presents the development of metal–polymer composite materials with the aim of application to micro-powder injection molding (µPIM) and metal material extrusion (MEX) for 3D printing. The research concentrates on developing a proper composition of materials, focusing mainly on the composition of the organic binder, deposition parameters, and debinding process. Commercial-grade iron powders were used, exhibiting high strength, toughness, conductivity, and low price, and therefore were considered an excellent alternative to other metal materials [27,28,29].

## 2. Materials and Methods

The technology related to micro-powder injection molding is more complex than classical powder metallurgy, and therefore, more processes and operations are involved. The main stages in the whole process are:

- powder selection and characteristics
- binder preparation
- mixing and homogenization of composition
- injection molding
- debinding
- sintering
- post-treatment (surface finish, etc.)

The materials for µPIM and MEX prepared for the experiments shared many similarities, and the main difference was in the process, whereby the injection molding is replaced with layer-by-layer free-forming, utilizing the classical FDM process.

### 2.1. Powders

**Figure 1:**
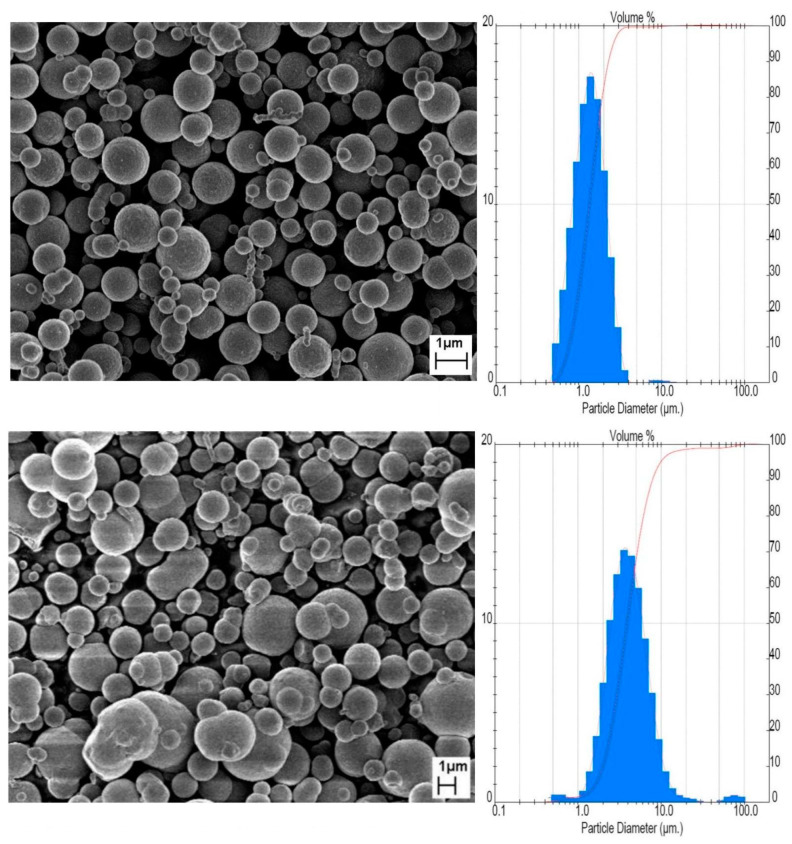
Scanning electron microscopy micrographs (**left**) and particle-size distribution (**right**) of the iron micro-powders used in the experiments: Fe_1_ top and Fe_4_ bottom. Blue bars represents percentages of particles with specific diameter and red line represents cumulative size distribution.

For the experiments, iron micro-powder provided by BASF (Ludwigshafen, Germany) was used. A detailed description of the materials used is listed in Table 1, along with SEM images and grain-size distribution plots, presented in Figure 1. The grain size was measured using the laser diffraction method with Mastersizer 3000 (Malvern Instruments Ltd., Malvern, UK). The powders had a spherical nature, as a result of the synthesis process whereby Fe(CO)_5_ decomposes to Fe and CO/CO_2_ gases and small residues of carbon. For the injection molding (PIM), both powder gradations were used, while for the additive molding (MEX), a powder with a grain size of 4 µm was used.

### 2.2. Binder

Selecting a proper organic binder is key to µPIM and MEX fabrication. The binder must have low viscosity, promote good dispersion of functional phase, and decompose above the dispensing temperature and below the sintering temperature, with very few residues. The detailed composition of the organic binder for PIM is presented in Table 2. Paraffin, as a main component, provided proper rheology at low temperatures, and was easy to remove with organic solvents. Carnauba wax and stearic acid improved the initial durability, preventing the shattering of injected samples. The low-density polyethene (LDPE) promoted cohesion of the samples after chemical debinding and through the sintering process, until the metal powders started to sinter. The composition introduced here was optimal for the µPIM fabrication of the components used in our previous unpublished work. Paraffin, carnauba wax and stearic acid were acquired from Flukar (Jasło, Poland), and LDPE was acquired from Synthos (Oświęcim, Poland).

The binder used for the µPIM technique can be easily formed into the shape of filament wires used in FDM, but after cooling to ambient temperature, they became hard and brittle, which is a significant limitation for this technique. During the printing, such brittle material blocks the printer’s extrusion system, because the fiber is fragmented on the drive rollers due to the pressure applied by them. Therefore, it was necessary to develop a binder with a similar decomposition method to that of the PIM binder, but still flexible after cooling. The composition of the organic binder for the polymer–metal composite for the FDM is presented in Table 2. Based on the composition of the PIM binder, a corresponding polymer composition was prepared and adjusted for the FDM technique. ABS was prone to chemical debinding, while LDPE was intended to maintain the shape after chemical debinding and decompose in the later thermal sintering process, as with the PIM technique. According to our unpublished studies and earlier experiments, ABS polymer tends to remain on the surface of the powders and other elements after dissolving with acetone. For most cases, it is impossible to remove the ABS entirely and therefore, by the rule of thumb, the final composition, being a mixture of ABS and LDPE, corresponds to the amount of polymer remaining after solvent debinding for the PIM technique. ABS was acquired from Finnotech (Katowice, Poland).

### 2.3. Composition Preparation

The mixing of organic binder components and homogenization of the final composition (with powders) was done during the same procedure. First, the binder components were heated to 125 °C in a twin Z-blade mixer LUK 015 (Werner & Pfleiderer, Stuttgart, Germany). After 30 min, metal powders were added, and the process continued for another 30 min. After that, the heating was turned off and, with rotating Z-blades, the mixture cooled and became dense, resulting in fine composite granules. In the case of the ABS–LDPE binder composition, it was necessary to achieve a mixing temperature of 200 °C (the plasticization temperature of the polymers) while the other mixing parameters were unchanged. Several composites were prepared with different volume fractions of metal powders (from 45 to 55 vol.%). The selection of the volume loading was based on the rheology measurement results and melt flow analysis for the corresponding forming techniques.

### 2.4. Rheology Measurements and Thermal Analysis

Rheology measurements of the obtained compositions were conducted on a heated, parallel-plate rheometer ARES from TA Instruments Inc., New Castle, DE, USA. It was crucial to perform the measurements at elevated temperatures to simulate the injection or extrusion process. Thermogravimetric analysis (TGA) and differential thermal analysis (DTA) were conducted on a thermal scale with the mass spectrometer STA 449 from Netzsch GmbH, Selb, Germany.

### 2.5. Injection Molding/Extrusion

**Figure 2:**
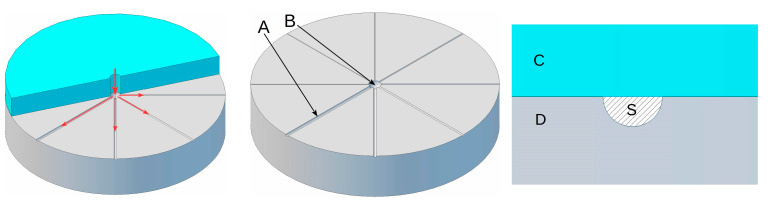
µPIM mold for flow test and simulation of extrusion through nozzles with different diameters: A—grooves simulating an extrusion fine-nozzle, B—sprue, C (blue) and D (gray)—upper and lower sections of mold, S—grooves cross-section. Red arrows indicate the material flow within the mold.

Injection-molding tests were performed on a constructed µPIM laboratory stand, modified from a standard injection-molding machine equipped with a heated powder chamber and injection mold, with a pressure in the range of 65–200 MPa. To test the material flow behavior in the fine nozzle, a special form with different diameters of the grooves was prepared (Figure 2) as a modification of the injection spiral flow test.

The fabrication of the elements in the MEX was carried out on an FDM Creality Ender-3 S1 Pro (Creality, Shenzhen, China) off-the-shelf printer. The tests were performed on nozzles of different diameters to determine the minimum values through which the material could be extruded. The test samples were made with the following printing parameters: nozzle temperature 190 °C, table temperature 90 °C, printing speed 10 mm/s, nozzle diameter 0.6 mm, layer height 0.2 mm, and 100% outline filling. For both procedures, rectangular prism samples were prepared, with external dimensions 20 × 8 × 4 mm for MEX and 10 × 2 × 2 mm for µPIM.

### 2.6. Debinding and Sintering

After the injection/extrusion of the composite material, a debinding process was conducted to remove the binder without shattering the element. In this case, chemical debinding was preferred because paraffin, stearic acid, and carnauba wax decomposed in several organic solvents, while a small amount of LDPE remained to provide cohesion of iron powders. The elements were immersed in tetrachloroethylene (Warchem, Warsaw, Poland) for five hours and left to dry in air. During the debinding process, samples were weighed every 30 min. The obtained samples were sintered at 1120 °C for 30 min, with 550 °C/20 min stop for the thermal debinding of the remaining LDPE. The process was conducted in 75%N_2_/25%H_2_ inert atmosphere.

Compared to PIM, for MEX it was necessary to change the solvent to acetone and perform the chemical debinding for 24 h. The ABS was subjected to chemical decomposition as the main component of the binder, while the LDPE acted the same way as with the µPIM approach, and remained to promote the cohesion of Fe powders, and later was decomposed during the thermal debinding and sintering.

## 3. Results

Composite material for MEX and µPIM should have appropriate rheology, be adapted to chemical and thermal debinding, and enabling obtaining sintered elements with applicable mechanical properties. These requirements make the development of materials and processes a challenging task. For these reasons, we needed to test several crucial material properties and process parameters during thermal analysis, rheology measurements, injection, debinding and sintering process analysis, and finally the mechanical tests.

### 3.1. Thermal Analysis and Rheology Measurements

**Figure 3:**
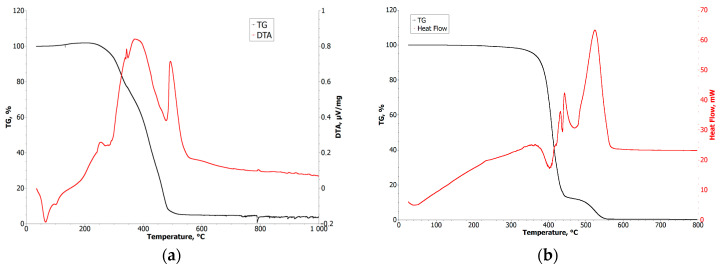
TGA and DTA curves of organic binder: (**a**) µPIM; (**b**) MEX.

To define the process parameters, it was crucial to examine the temperature behavior of the organic binders. The results obtained from the analysis enabled the design of a temperature profile for the thermal debinding and sintering process. Crucial points to define were related to the decomposition and evaporation of the organic binder elements. In Figure 3, the results of the TGA/DTA analysis are presented. We can observe that the complete decomposition of LDPE, being the most thermally resistant component of organic binder, is around 520 °C.

The differences between the binders used were mainly in the decomposition temperatures. In the case of material containing paraffin, decomposition could be carried out both by solvent and thermal methods because the temperature range was quite broad, and part of the binders (paraffin) began to evaporate before the LDPE began to plasticize. This enabled maintaining the integrity of the shape. In the case of the MEX binder, both materials had similar softening and decomposition temperatures, which forced the use of solvent decomposition initially and then burning off the polymer residues in the sintering process at a temperature above 550 °C.

**Figure 4:**
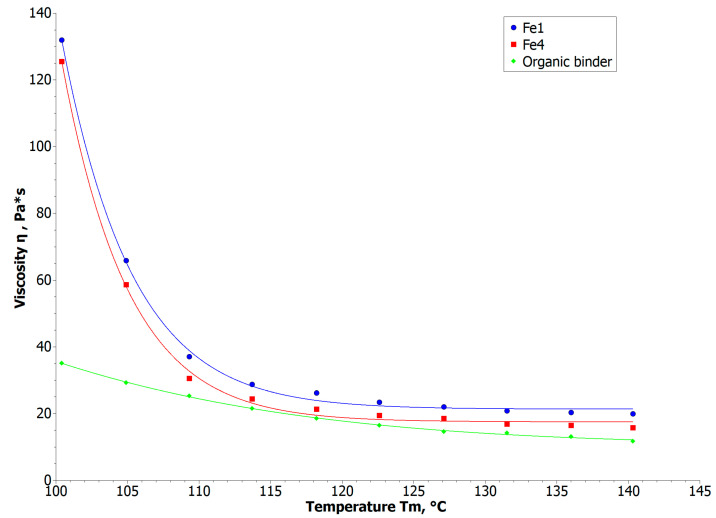
Rheology investigation: a function of viscosity value influenced by temperature change for µPIM compositions.

A complete analysis of temperature-dependent rheology for compositions filled with 60 vol.% of Fe_1_ and Fe_4_ is presented in Figure 4. We can observe that the viscosity of the composition is highly related to temperature change up to 110 °C, afterward remaining stabilized and not temperature dependent. On this basis, the injection temperature was set at 125 °C.

Similar results were obtained for the ABS–LDPE binder regarding the viscosity decrease with temperature rise. The order of magnitude of the data was different for both materials presented. At temperatures above 150 °C, LDPE and ABS began to reduce their viscosity, and the paraffin-based binder decomposed rapidly at this temperature.

### 3.2. Injection/Extrusion Process Analysis

**Figure 5:**
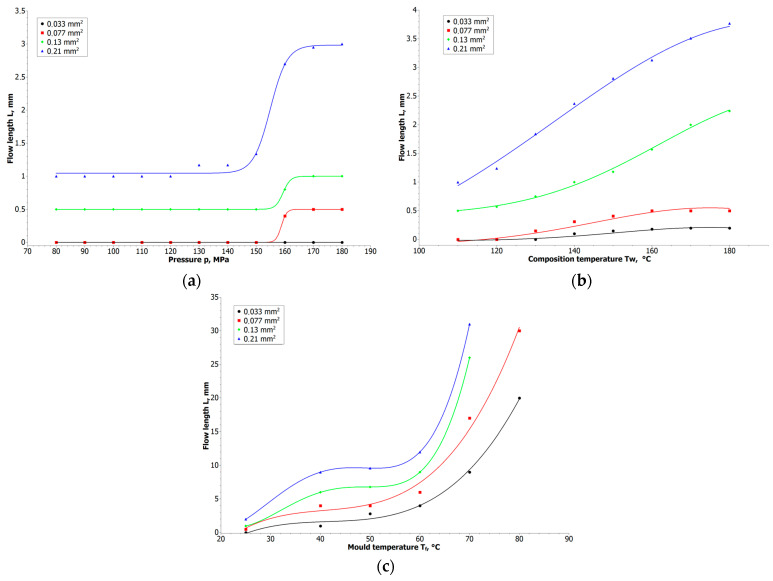
Influence of the injection process parameters on flow length in the function of: (**a**) injection/extrusion pressure; (**b**) composition temperature; (**c**) mold temperature.

In the case of the injection/extrusion process, several parameters needed to be examined: injection/extrusion pressure, mold/nozzle temperature, and the diameter of the mold grooves or nozzle. Therefore, we have conducted three types of experiment of injecting compositions under different pressures, with varying mold temperatures and for different diameters of grooves in mold. The result was the flow length of the composition. This parameter was chosen to evaluate the performance of the prepared composites in the extrusion process. To obtain the fine-detail elements, we were forced to use fine extrusion nozzles, therefore we needed to determine if the material was able to pass through the small-diameter nozzle without a high process pressure and eventually avoid clogging the nozzle. Figure 5 presents the results from these three experiments. We can observe that for the small groove diameter, the pressure did not influence the flow length, which remained at a low level, and only for the high cross-section area (above 200 µm) did we observe total filling of the groove. A more substantial influence on the flow length of the material was the temperature. We could observe distinct changes in the flow length above 60 °C, which is above the melting temperature of paraffin and close to those of wax and stearic acid. It had a strong influence on lowering the viscosity of the composition. The temperature of the mold also had a strong impact because, for lower temperatures, we observed a shorter flow length, related to the cooling of the material and an increase in viscosity.

Table 3 contains the parameters needed to extrude the material through the printing nozzles of various diameters in the MEX technique. The extrusion temperature of pure ABS recommended by the manufacturer is 230 °C, but we were unable to reach that value, while at 210 °C, the nozzle became clogged for most materials and nozzle diameters. On the other hand, at temperatures below 190 °C, the material plasticized so poorly that the printed layers had no adhesion to each other. Finally, for the 55 vol.% fillings, it was impossible to obtain the correct element, even with the largest nozzle diameters, regardless of the printing temperature.

### 3.3. Debinding and Sintering

**Figure 6:**
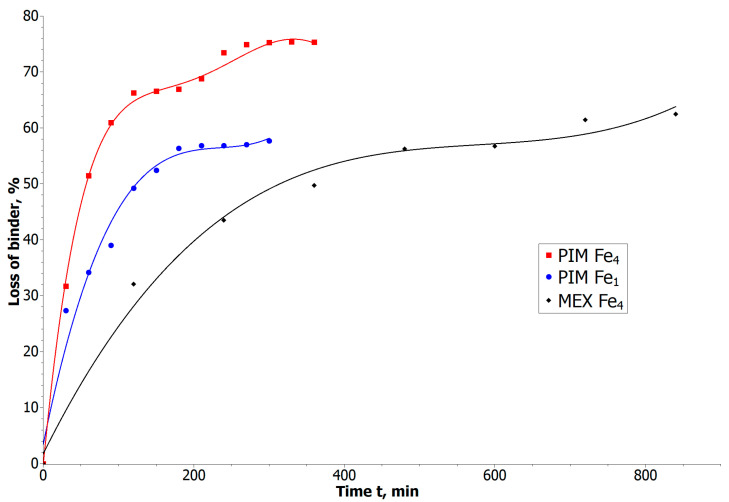
Debinding process characteristic for the obtained composite elements, with the same volume of the iron micro-powders (50%).

Chemical debinding for the µPIM was performed with tetrachloroethylene to dissolve all binder components except LDPE. During debinding, the structure became porous. Contorted porosity is favorable for the evacuation of LDPE residues during thermal decomposition. In Figure 6, we present the time-dependent dissolving of binder for Fe_1-_ and Fe_4_-filled composites. In the first stage of the process, we can observe faster dissolving of material located on the surface of samples, but after 1.5 h, the material loss is barely related to the process time, while penetration of solvent inside the elements is limited. We can see a strong dependence on the powder size (and thus porosity) on the volume loss of the binder.

In the case of the ABS–LDPE binder, the chemical debinding was carried out using acetone. Due to the size of the samples, the time needed for the whole process increased to 24 h. During the first 8 h, the process ran quickly and then slowed down, which is apparent in the weight loss of the samples.

After chemical debinding, samples were heated in the oven to 550 °C to perform thermal debinding of remained LDPE. After that procedure, the temperature was increased to 1120 °C to perform sintering of the iron powders. Sintered samples were durable, that allowed performing further tests.

### 3.4. Shrinkage

Determining the dimensional accuracy and shrinkage of the elements after sintering required a series of measurements after the shaping process (injection or printing), and then repeating the measurements after sintering. The results after sintering are presented in Table 4. In the case of the PIM, the dimensions of the samples were compared against the dimensions of the injection mold geometry, while the 3D-printed MEX samples were measured after the printing process and sintering.

## 4. Discussion

The rheology and thermal effects observations are the starting point for manufacturing parameters in the fine-nozzle extrusion process. In most cases, the binders used in the PIM were mixtures of several organic components selected for a efficient injection molding, debinding and sintering processes [30,31,32]. In the low-temperature stage, paraffin and waxes are removed, and in the high-temperature stage, polymers are decomposed just before the sintering of metal powders. As presented in Figure 3a, the DTA curve fits the thermal properties of the organic binder. In the first region, we can observe two thermal changes: at 65 °C, associated with the melting of paraffin and stearic acid (total 76% of binder); at 102 °C, associated with wax melting and LDPE melting and oxidizing—confirmed by a 2% increase of weight at TGA curve. At 251 °C (DTA curve) and 233 °C (TGA curve), we can observe the decomposition of organic components. These observations suggest that the process temperature should be kept above the melting point of each component and below the threshold of negative thermal influence on the binder (from 100 °C to 200 °C). These results correspond to the rheology measurements presented in Figure 4 and Figure 5c, where significant changes in viscosity are seen up to 110 °C and significant flow length is observed above 60 °C. Therefore, a material temperature—the first parameter of the injection or extrusion process—should be kept close to 110 °C.

**Figure 7:**
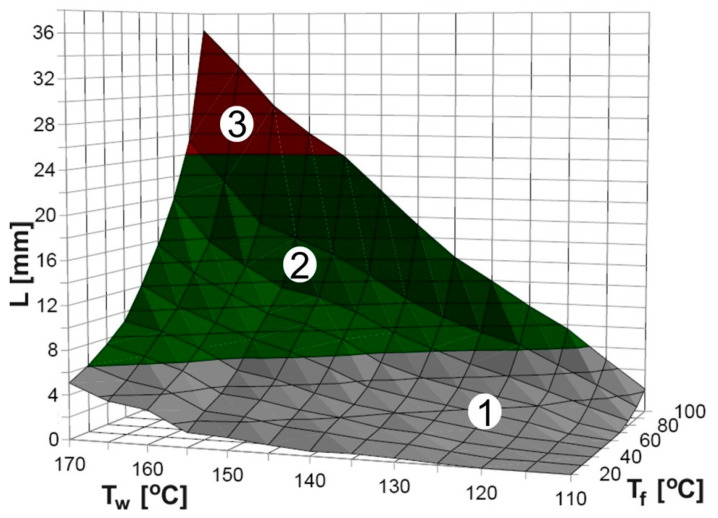
Process window visualization with optimal temperature regimes: first region (gray)—clogging, second region (green)—optimal, third region (red)—degradation of organic binder. Notes: T_f_—mold temperature, T_w_—material temperature.

The temperature of the nozzle, simulated by the temperature of the mold, influences directly the flow length of the material (Figure 5b). More efficient wetting of the nozzle walls [33] will allow efficient deposition of the material and will prevent clogging. Along with the TGA/DTA results, this allowed us to propose a process window for optimal temperature regimes, presented in Figure 7. While the injection pressure has a modest influence on the obtained results, and the cross-section area of the groove (nozzle diameter) has more influence on the geometry of the elements than on the extrusion process, we have distinguished three regions related to the influence of the material and mold temperature on the flow length:

- the first region (gray) corresponds to the insufficient flow length, resulting in the clogging of the fine-extrusion nozzle,
- the second region (green) is optimal for the extrusion of the material,
- the third region (red), with a very impressive flow length, is dominated by too high a temperature, causing degradation of the organic binder. The high temperature will cause geometric deformations in the final printed elements, as well as if the material stays for a long time in a low-viscosity state.

In the case of the MEX molding, the feasibility is influenced by powder filling, nozzle temperature, and nozzle size. The smaller the powder filling, the lower the viscosity of such material [34], and therefore it will flow better through the nozzle. However, when trying to implement large filling volumes of the powder, there is a problem with the material flow. One observation during the test was a substantially large lump of plasticized material in the entrance to the nozzle, making the extrusion impossible. Thermal softening of the composite filament reaches near the rolling gears of the feeding system. A corresponding phenomenon was observed for the PIM process for a small cross-section of the grooves, in which the material cooled very quickly and could not be injected in the entire volume (Figure 5c). In both cases, this was related to the thermal conductivity of the composite material containing large volume of the metal powder. The solution for this problem is introducing a larger nozzle diameter for the MEX technique, to ensure faster flow and distribution of the filament in the heated nozzle and, by analogy, implementation of a larger cross-section of the channels for the PIM technique to allow a better flow of the injected material.

**Figure 8:**
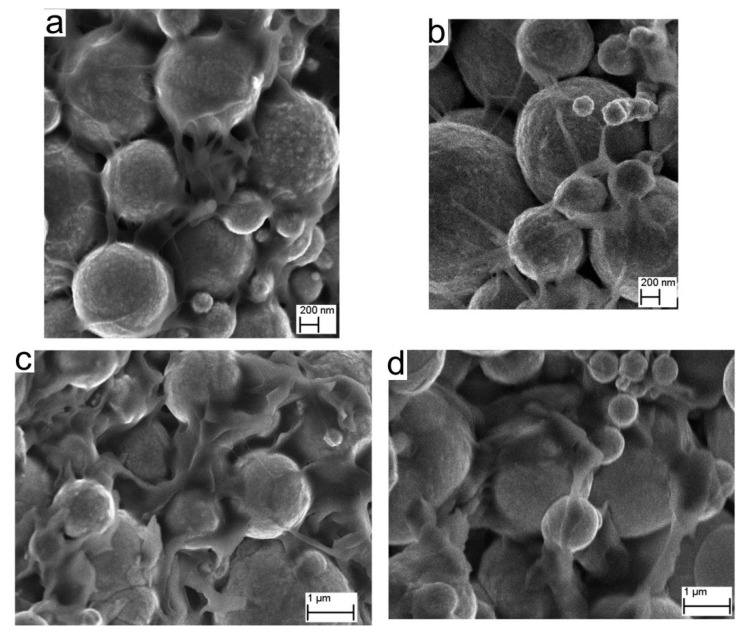
Scanning electron microscopy micrographs of samples after the chemical debinding process, with 50 vol.% of the powders: (**a**) Fe_1_ after 60 min and (**b**) 180 min; (**c**) Fe_4_ after 30 min and (**d**) 60 min.

The results of the chemical debinding process (Figure 6) indicate a strong influence of porosity on the debinding efficiency. The dissolving of the binder components is limited for elements filled with smaller powder grains (Fe_1_). Scanning electron microscopy (SEM) observations of cross-sections of the samples after a specified time of debinding (Figure 8) reveal that the longer the process is maintained, the more binder is removed, although LDPE insoluble in tetrachloroethylene remains. These bridges of LDPE bind the powder together and preserve the structural integrity before the sintering process.

**Figure 9:**
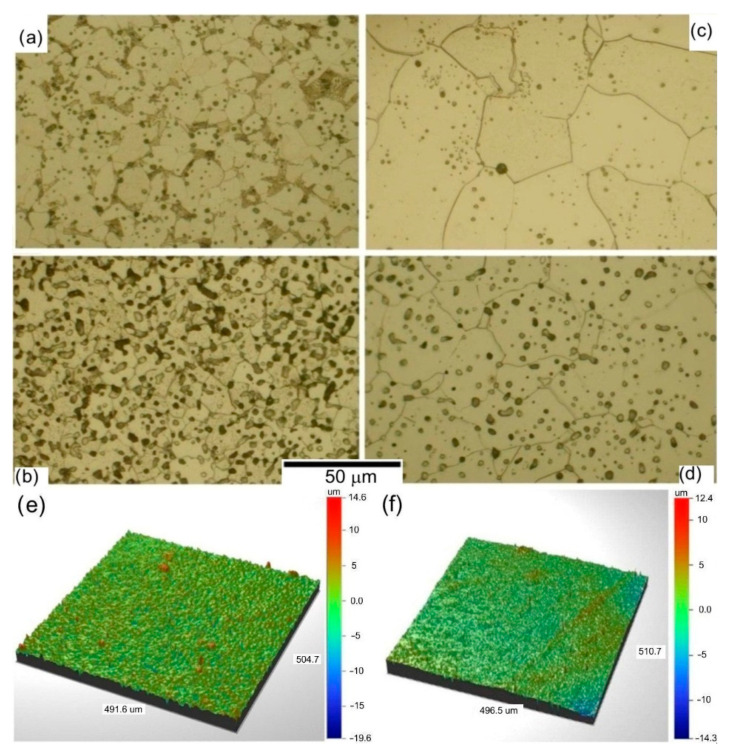
Microstructure of sintered samples with different iron powders and volume loadings: (**a**) Fe_1_ 55 vol.%, (**b**) Fe_1_ 60 vol.%, (**c**) Fe_4_ 55 vol.%, (**d**) Fe_4_ 60 vol.%. Surface roughness profiles of sintered samples: (**e**) Fe_4_ 55 vol.%, (**f**) Fe_1_ 60 vol.%—(measured with non-contact optical profiler WYKO NT2000 from Veeco Instruments Inc., Plainview, NY, USA).

After injection forming or printing of the samples and chemical debinding, heat treatment is carried out to remove the LDPE through thermal debinding and afterwards sintering the iron powders. In this case, TGA/DTA analysis reveals that the decomposition of the organic binder starts at 250 °C with significant weight loss, and ends at 524 °C where weight loss is negligible (5 wt.% of binder residues remains). Therefore, the final process was conducted in two steps: 550 °C for the thermal debinding and 1120 °C for the iron powders sintering. Inside the sintered samples, we can observe residues of organic binder. The amount of residue is related to porosity—a function of grain size and volume loading. In Figure 9, we can observe that for samples with higher loading (Figure 9b—Fe_1_ 60 vol.%, Figure 9d—Fe_4_ 60 vol.%) and samples with smaller powder grains (Figure 9a,b Fe_1_), there are more impurities caused by organic binder decomposition; more than for corresponding samples with lower volume loading or samples with larger grain size. Here, we can observe signatures of the reactions between decomposing polymer and Fe powders as darker regions on the grain boundaries and as spots, signalizing the occurrence of carbide formation. The evacuation of the decomposition residues is severely limited by low porosity in the samples.

To evaluate the porosity change among the samples, the indirect approach was undertaken, with surface roughness measurements of the samples with different volume loadings (Figure 9e,f). A distinct difference in surface roughness is observed between S_a_ = 1.38 µm for Fe_4_ 55 vol.% sample (high diameter, low volume loading) and S_a_ = 0.63 µm for Fe_1_ 60 vol.% sample (low diameter, high volume loading), respectively, suggesting that samples with higher volume loadings exhibit lower porosity.

Interestingly, although it was necessary to change the binder between PIM and MEX, the shrinkage of the samples for the same grain size is similar. Table 4 shows that for filling 45 vol.% and 50 vol.% with Fe_4_ powder, the results are comparable for both techniques and materials [35]. In the case of the PIM, higher fillings are achieved, which reduces the shrinkage of elements. We want to obtain relatively repeatable dimensions of parts and correct shape reproduction, so significant shrinkage is not desirable. Another solution is to use finer powders, which leads to more significant shrinkage, but a finer powder allows for better detail reproduction. Also, using larger fillings and finer powders results in fewer pores in the finished element [36].

## 5. Conclusions

The paper describes a comprehensive approach to fabricating sintered metallic elements with micro-powder injection molding (µPIM) and metal material extrusion (MEX) targeted on 3D printing. An original composition based on iron micro-powders and organic binder mixture was developed. The binder formulation consisted of several organic components, including paraffin, LDPE, wax and stearic acid, with content precisely adjusted to exhibit rheological and thermal properties suitable for MEX and µPIM applications, where micro-extrusion and micro-injection occur. We have proposed a procedure similar to the popular injection spiral test to analyze the composition flow in the micro-channels, which corresponds to the heated fine-nozzle in MEX. The optimal process conditions were determined, such as injection/extrusion pressure, diameter and temperature of the nozzle or mold groove, and temperature of the composite material. We have observed that clogging occurs with a small diameter, regardless of the pressure, while the predominant factor is the temperature of the material and mold/nozzle. A distinct change in flow length was observed above 60 °C, related to a decrease in the viscosity value of the composition. A high influence of the material’s porosity—a consequence of the powder diameter—was observed. The dissolving of the binder components is limited for elements filled with smaller powders, but an additional thermal debinding process allowed preparing samples for sintering. Additional chemical analysis of the exhaust gases from the thermal decomposition and sintering process would also be very useful to evaluate the differences between the used polymer binders and their influence on the final sintered parts. The obtained sintered elements were of high quality and showed minor signs of binder-related flaws after sintering. Further experiments need to be performed, with a focus on the use of metal nano-powders for lower sintering temperature and fabrication of fully MEX 3D printed elements, with the perspective of application in structural electronics as electrically and thermally conductive paths. Also, incorporating the evaluation of mechanical properties would allow a more comprehensive evaluation of the quality of the elements fabricated with such techniques and their practical applicability.

## Figures and Tables

**Table 1:** Properties of iron micro-powders used for experiments.

Powder	Density	Particle Size: Catalog (Measured)
Fe_1_	7.6 Mg·m^−3^	1 µm (1.35 µm)
Fe_4_	7.5 Mg·m^−3^	4 µm (3.89 µm)

**Table 2:** Organic binder composition.

Material		Type/Grade	Melting Point	Content
µPIM	Paraffin	R II	54 °C	70 vol.%
	LDPE	Malen E	115 °C	16 vol.%
	Carnauba wax	T1	82 °C	8 vol.%
	Stearic acid	ST II	71 °C	6 vol.%
MEX	ABS		94 °C	80 vol.%
	LDPE	Malen E	115 °C	20 vol.%

**Table 3:** Material flow depending on the temperature and nozzle diameter for the MEX extrusion technique. Proper deposition is indicated by the “+” sign, and failure is indicated as “−” sign.

	Material (Fe)	45 vol.%			50 vol.%			55 vol.%
	Nozzle (mm)	1.0	0.8	0.6	1.0	0.8	0.6	1.0
Temperature [°C]	180	−	−	−	−	−	−	−
	185	+/−	+/−	+/−	+/−	+/−	+/−	−
	190	+	+	+	+	+	+	+/−
	195	+	+	+	+	+	+	−
	200	+	+	+	+	+	+/−	−
	205	+	+	+/−	+/−	+/−	−	−
	210	+/−	+/−	−	−	−	−	−

**Table 4:** Shrinkage values calculated for the injection molded and 3D-printed samples after the sintering process.

Powder Filling	PIM Fe_1_	PIM Fe_4_	MEX Fe_4_
45 vol.%	15.87%	14.88%	15.88%
50 vol.%	14.08%	13.78%	13.93%
55 vol.%	12.86%	12.09%	-
60 vol.%	11.69%	10.93%	-

## Data Availability

The data presented in this study are available on request from the corresponding author.
